# How can health remain central post-2015 in a sustainable development paradigm?

**DOI:** 10.1186/1744-8603-10-18

**Published:** 2014-04-03

**Authors:** Peter S Hill, Kent Buse, Claire E Brolan, Gorik Ooms

**Affiliations:** 1 School of Population Health, The University of Queensland, Herston Road Herston, 4006 Brisbane, Australia; 2 Joint United Nations Programme on HIV/AIDS, (UNAIDS), 20 Avenue Appia CH-1211, Geneva 27, Switzerland; 3 Institute of Tropical Medicine, Nationalestraat 155, 2000 Antwerpen, Belgium

**Keywords:** Millennium Development Goals (MDGs), Sustainable Development Goals (SDGs), Sustainable development, United Nations, Universal health coverage, Non-communicable diseases, Sexual and reproductive health and rights, Health policy

## Abstract

In two years, the uncompleted tasks of the Millennium Development Goals will be merged with the agenda articulated in the 2012 United Nations Conference on Sustainable Development. This process will seek to integrate economic development (including the elimination of extreme poverty), social inclusion, environmental sustainability, and good governance into a combined sustainable development agenda. The first phase of consultation for the post-2015 Sustainable Development Goals reached completion in the May 2013 report to the Secretary-General of the High-Level Panel of Eminent Persons on the Post-2015 Development Agenda. Health did well out of the Millennium Development Goal (MDG) process, but the global context and framing of the new agenda is substantially different, and health advocates cannot automatically assume the same prominence. This paper argues that to remain central to continuing negotiations and the future implementation, four strategic shifts are urgently required. Advocates need to reframe health from the poverty reduction focus of the MDGs to embrace the social sustainability paradigm that underpins the new goals. Second, health advocates need to speak—and listen—to the whole sustainable development agenda, and assert health in every theme and every relevant policy, something that is not yet happening in current thematic debates. Third, we need to construct goals that will be truly “universal”, that will engage every nation—a significant re-orientation from the focus on low-income countries of the MDGs. And finally, health advocates need to overtly explore what global governance structures will be needed to finance and implement these universal Sustainable Development Goals.

## Background

With less than two years until the 2015 Millennium Development Goals’ (MDG) deadline, progress towards the achievement of the global targets presents a mixed picture. Goals addressing extreme poverty have been met; hunger reflected in under-nutrition should be halved; 2.1 billion people have gained access to improved sources of drinking water. Unprecedented gains have been made in combating malaria and tuberculosis, and providing access to anti-retroviral medicines [1]. Despite progress in the reduction of preventable infant and maternal mortality, limited access to antenatal care and skilled birth attendance remains problematic in rural areas, particularly in sub-Saharan Africa [1]. Widespread regional disparities and rural–urban gaps persist. There is clearly unfinished work from the MDGs to be carried forward into the next iteration of global development: fragile and conflict-affected states are least likely to achieve their goals, and most vulnerable in a transition to new goals [2]. Yet “The World We Want”—the vision of the post-2015 development goals—speaks to much more than the completion of the MDG agenda [3].

### Health in the UN consultations on the post-2015 goals

The consultation phase for the Post-2015 Goals has been extensive. Since September 2012, the UN agencies have coordinated 11 global thematic and 88 national consultations, collating submissions from development agencies, civil society, academia, governments—even web-based personal submissions. The Global Thematic Consultation on Health, in its report “Health in the Post-2015 Agenda”, [4] identified “maximizing healthy lives” as its goal to link health to sustainable development, with three targets:

• “Accelerating progress on the health MDG agenda” reaffirms and extends the three MDG goals, incorporating neglected tropical diseases (NTDs) as an additional focus;

• “Reducing the burden of major Non-Communicable Diseases” (NCDs), addresses cardiovascular diseases, cancers, chronic respiratory diseases, diabetes and mental health—conspicuously absent from the MDGs;

• “Ensuring universal health coverage and access”, strongly advocated by World Health Organization (WHO), was proposed as an operational framework for the health targets [4].

These recommendations were largely accepted in the report to the UN Secretary-General of the High-Level Panel of Eminent Persons on the Post-2015 Development Agenda, presented in May 2013 [5]. Health was retained among their “illustrative goals”; the health MDGs have been reconfigured, the NCD agenda is included—though considerably weakened [6]—and “universal sexual and reproductive health and rights” has now gained prominence as a target. Universal Health Coverage (UHC) has been incorporated, not as a target, but as instrumental to achieving health outcomes. Under a single proposed health goal, all elements of the three health-goal MDG position have been maintained—and with the inclusion of NTDs and NCDs, significantly extended [5].

### Two tracks to 2015: poverty reduction and sustainable development

But even before the completion of the UN consultation, a parallel process had commenced, based on the agenda articulated in the 2012 United Nations (UN) Conference on Sustainable Development (Rio + 20) [7]. Rather than focusing only on poverty reduction in low- and middle-income countries, the Sustainable Development Goals (SDGs) envisaged at that conference were conceived as global, addressing the global implications of development for all states, and integrating economic development (including the elimination of extreme poverty), social inclusion, environmental sustainability, and the good governance that ensures peace and security [8]. Carriage of the planning for these post-2015 SDGs has shifted from the UN agencies to the Member States: the Open Working Group on SDGs will report its findings to the UN General Assembly this year. UN Secretary-General Ban Ki-moon has called for the two processes to converge, with a final synthesis of Post-2015 Goals to be endorsed at the UN General Assembly in September 2015, arguing that this presents a critical opportunity to produce ambitious, yet realistic, post-2015 development outcomes: “a daunting yet inspiring and historic task [9]”.

### Health in the sustainable development agenda

Health advocates cannot be presumptuous about how health will be configured in the final goals, or their implementation. Its omission would be highly unlikely: the recent “Progress report of the Open working Group of the General Assembly on Sustainable Development Goals” notes that “Health is a right and a goal in its own right, as well as a means of measuring success across the whole sustainable development agenda” ([10], p.13). But concerns have been voiced that despite inclusion in the SDGs, the same level of generosity of the MDGs may not be extended to health again, having “had its moment in the spotlight and succumbing to competition from other issues demanding attention, such as climate change or food security [11]”. The strength of the final report of the High-Level Panel of Eminent Persons did not lie in its itemization of the illustrative goals: each goal—health included—was relegated to the annex and allocated just two pages. The highlight of that report is its five transformative shifts, central to their vision of change, with a focus on equity, sustainable development, economic growth and jobs, peace and governance and a new global partnership [5]. The Open Working Group [10] and the Sustainable Development Solutions Network both include health as a theme, [12] but recent publications demonstrate how health could be readily subsumed within other SDG framings [8,13].

If health is to retain prominence in the post-2015 goals, global health strategists need to be aware of the substantial difference in context that the sustainable development agenda represents. The key reports tabled to date suggest consensus over the options, though there is some question around the preferred hierarchy and relationships between goals, targets and the means of achieving them. But the MDG focus on extreme poverty, with its particular emphasis on health, now needs to be reconciled with new and competing priorities. With climate change and environmental degradation threatening global growth and security, the international development community needs to prioritize “integrating environmental sustainability into its architecture and actions [14]”. While the work of the MDGs in transforming low-income countries will need to be continued, and extended to meet the basic needs of the billion poor in middle-income countries, the SDGs will make universal claims: economic development, environmental sustainability and social inclusion are global issues. And health will be included only in so far as it can be demonstrated to be integral to each of these, to respond to the spectrum of sustainable themes that are currently being explored. To achieve this, four strategic shifts are urgently required.

### Re-orientating health in the sustainable development process

#### Reframing health in terms of social sustainability

First, advocates need to recognize the need to transform their framing of health from the poverty reduction paradigm of the MDGs into the social sustainability paradigm of the SDGs. “Achieving health and wellbeing at all ages”—the proposed health SDG—requires new perspectives: an emphasis on health rather than disease, effectively defining “wellbeing” and reimagining health goals as truly universal. The proposal by The Lancet Commission on Investing in Health for a “grand convergence” offers a comprehensive but feasible bid to target the inequities of global health [15]. Under-5 mortality and annual death rates for AIDS and tuberculosis stand as the sentinel targets, but act as proxies for systemic change. The proposal reiterates the central message of the 1993 World Development Report [16]. It addresses the economic dimensions of sustainable development by arguing for the “enormous payoff from investing in health” and outlining potential efficiencies, mobilizing financing options and ensuring financial protection and equity through progressive Universal Health Coverage [15]. The institutions needed to ensure that these gains are maximized—institutions for adequate information, civic deliberation, efficient finance, just stewardship, ensuring normative standards and guidelines, and independent accountability—speak to the governance that integrates the three “pillars” of sustainable development [17]. Yet the strategies are essentially sectoral in nature, and the challenge of “tackling the social and intersectoral determinants of health” has been sidestepped as too political or protracted [15]. But reintegrating this economic discourse into the health debate in the context of sustainable development would open up opportunities to engage those social determinants, and deliver on the synergies available from social sustainability.

#### Speaking and listening to the whole sustainable development agenda

But this requires that health advocates speak—and listen—to the whole SDG agenda, and assert health in every theme and every relevant policy. In their analysis of the links in the UN consultation documentation on each of the 11 post-2015 themes, Kickbusch and Brindley [18] note that health is “talking to” and being “heard” by a limited number of themes—sustainability, water, food and inequalities—but is not connecting well with the remainder. Where other thematic consultation reports refer to health, they construct it narrowly in terms of physical and mental health, less frequently referring to health systems, risk factors, and the structural and social determinants of health, including enabling legal environments, financing and governance. Given that health is largely determined by social and environmental factors, should health champions not also highlight the interdependence of health and the themes of education, growth, population, energy and indeed governance? And given WHO’s declared commitment to UHC, should they not also be pointing to how UHC contributes to better governance and the building of sustainable social structures—particularly in contexts where these are eroded by conflict and fragility? [2]. In the SDG vision of the future, advocates need to ensure that health is not merely an indicator for social sustainability—that economic, social and environmental sustainability create health and wellbeing—but that the active protection of the health and well-being of a population, is integral to its social, economic and environmental sustainability.

#### Constructing universal health goals

Third, advocates need to construct goals that will engage every nation—as the “universality” of the SDGs suggests—and that will challenge the contribution of every nation to global change. This will inevitably introduce a tension of priorities between countries in different states of economic development. The MDGs mobilized solidarity, but with low- and middle-income countries as the principal focus: high-income countries were motivated to increase their development assistance, sharing the financial and technical burden of achieving the goals in low-income countries, but did not feel bound to apply the demands of MDG measurement to their own performance. Yet despite overall global progress, there are persisting challenges in least developed countries, and health and economic inequalities in middle- and high-income countries have simultaneously increased significantly [19]. The remaining MDG burden, the globalizing drivers of the NCDs (poor diet, alcohol and tobacco, sedentary lifestyles, the erosion of social networks) and the politics of sexual and reproductive health and rights—together these will ensure that in the coming two years all member states will need to engage the challenge of formulating SDGs that will impact on them – and addressing the complex associated political challenges thereof [6]. With its goal of “health and wellbeing at all ages”, the SDGs will need a dual strategy: each member state addressing the global goals; all member states united in global solidarity.

#### Envisioning the global governance structures for this new paradigm

Finally, if health is to maintain its central role as the MDGs are folded into the SDG agenda, advocates need to overtly explore what global governance structures will be needed in this new paradigm. The turn of the Millennium provided a context for the burgeoning of what Kickbusch and Szabo define as *global health governance*—“those institutions and processes of governance with an explicit health mandate” [20]—adding new institutions such as the GAVI Alliance and the Global Fund to Fight AIDS, Tuberculosis and Malaria (GFATM) to the existing UN agencies, and new mechanisms such as the Framework Convention on Tobacco Control and the revised International Health Regulations. But the SDGs demand an increasing interface with *global governance for health*—those institutions and processes that directly and indirectly impact on health in the context of globalized trade, security, migration and the environment [21]—and governance structures that will see health interface across the whole sustainable development agenda.

The Lancet-University of Oslo Commission on Global Governance for Health [21] proposes a UN Multistakeholder Platform on Global Governance for Health, functioning as a policy forum, rather than a funding platform. Their proposed Independent Scientific Monitoring Panel on Global Social and Political Determinants of Health would deploy the “best minds to investigate the complex interaction of forces that lead to health outcomes”, [22] but neither makes the connection between the governance and funding that will be pivotal to achieving the global solidarity that Frenk et al. see as imperative in this era of complex interdependence [11].

The success of the Global Fund has not gone unnoticed as a model for collaborative global funding: an expanded Global Fund for Health has been proposed, [23] as has a Framework Convention on Global Health, [24] each reliant on a global partnership that would commit to underwrite a global provision of essential health services. But beyond health, a Green Climate Fund to assist developing countries in mitigating the effects of climate change has been proposed out of the Copenhagen accord. With a budget targeting US$100 billion annually by 2020, it would eclipse current projections for the global health institutions [25].

The funding for the Green Climate Fund is intended to be additional to development assistance, though additionality will be difficult to measure in the context of competing demands for post-2015 resources. For the European Commission, “a comprehensive and integrated approach to financing poverty eradication and sustainable development” is the preferred option, [26] and it seems likely that the Green Climate Fund will play a key role in this integrated approach.

The debates around the post-2015 health goals have not prioritised the potential impact of climate change on health needs, particularly in least developed economies [27]. This failure to anticipate may be costly for health interests: the belated “discovery” of climate related health impact on health systems may arrive too late to gain prominence in the Green Climate Fund priorities; the global health institutions do not have a good record of responsiveness or flexibility in working together on health systems support, [28] and an additional financing mechanism for health that is limited to the impact of climate change may not be an acceptable solution.

But the time may be right for a larger vision, if health advocates are able to relocate health into the new paradigm, to present it as a precondition for social sustainability, as a precondition for “public concern for the state of the natural environment”, and necessarily linked to the progress to prosperity [29]. The insularity of current health sector advocacy is in tension with the recognition that health is integral to sustainable development, and that sustainable development is essential for health. For health to remain central in the post-2015 Sustainable Development Goals, it will need to be pervasive in each of the dimensions of sustainable development—economic, social, environmental—not quarantined from them. Engaging the new sustainability paradigm offers an unprecedented opportunity. A ‘green and social environment fund’ could be the answer to comprehensive and integrated approach desired by the European Commission and others; one in which health figures prominently and pervasively. That could realize the “daunting yet inspiring and historic” task that Ban Ki-moon refers to.

## Abbreviations

MDG: Millennium Development Goal; NCD: Non-communicable disease; NTD: Neglected tropical disease; Rio + 20: United Nations Conference on Sustainable Development; SDG: Sustainable Development Goal; UN: United Nations; WHO: World Health Organization.

## Competing interests

The authors declare that they have no competing interests.

## Authors’ contributions

PSH, CEB and KB conceived the paper; PSH completed the first draft. KB, CEB and GO have revised the draft critically for content and analysis. All authors agree to the final content, and take responsibility for it. 
